# Dysphagia Secondary to Extrinsic Esophageal Compression From an Anterior Mediastinal Squamous Cell Carcinoma

**DOI:** 10.7759/cureus.109053

**Published:** 2026-05-17

**Authors:** Alireza Izadian Bidgoli, Alberto Gomez Veliz

**Affiliations:** 1 Internal Medicine, American University of the Caribbean School of Medicine, Cupecoy, SXM; 2 Internal Medicine, Jackson Memorial Hospital, Miami, USA

**Keywords:** dysphagia, extrinsic esophageal compression, mediastinal mass, nondiagnostic biopsy, squamous cell carcinoma

## Abstract

Dysphagia is commonly attributed to intrinsic esophageal pathology; however, extrinsic compression from mediastinal processes remains an important and often underrecognized cause. We report the case of a 69-year-old man who presented with acute dysphagia due to food impaction. Esophagogastroduodenoscopy successfully relieved the obstruction but revealed no intrinsic lesion, instead demonstrating extrinsic compression of the upper esophagus. Subsequent cross-sectional imaging identified a large anterior mediastinal mass extending into the left neck, encasing the aortic arch and its branches, and causing significant esophageal deviation and stenosis. Laboratory evaluation was notable for leukocytosis, anemia, elevated inflammatory markers, and an elevated carcinoembryonic antigen level. Initial attempts at tissue diagnosis, including ultrasound-guided axillary lymph node sampling and endobronchial ultrasound-guided transbronchial needle aspiration, were nondiagnostic due to insufficient or nonrepresentative tissue. Given persistent high clinical suspicion, further tissue sampling was pursued, ultimately establishing a diagnosis of squamous cell carcinoma. This case highlights the diagnostic limitations of endoscopy in the evaluation of extraluminal disease, the challenges of tissue acquisition in mediastinal pathology, and the critical importance of persistence following nondiagnostic biopsies. An imaging-driven, multidisciplinary approach is essential for accurate diagnosis and timely management in patients with suspected mediastinal malignancy.

## Introduction

Dysphagia is a common clinical complaint most frequently attributed to intrinsic esophageal pathology, including structural lesions and motility disorders [1,2]. However, extrinsic compression of the esophagus by mediastinal processes represents an important but often underrecognized etiology [3]. Mediastinal masses comprise a heterogeneous group of benign and malignant conditions, with clinical manifestations largely dependent on size, location, and involvement of adjacent structures [4]. Esophageal compression from these lesions may lead to progressive or acute dysphagia and may occasionally present as food impaction or obstructive symptoms despite the absence of primary intraluminal esophageal disease [2,3].

Malignancies involving the mediastinum include lymphomas, thymic epithelial tumors, germ cell tumors, and metastatic disease [4,5]. Squamous cell carcinoma (SCC), most commonly arising from the lung or upper aerodigestive tract, may present as a mediastinal mass through direct extension or nodal metastasis and can manifest with compressive symptoms in the absence of overt mucosal involvement [5,6]. This creates an important diagnostic blind spot, as initial upper endoscopy may demonstrate luminal narrowing or nonspecific external compression without identification of a visible intraluminal lesion, potentially leading to underrecognition of an underlying mediastinal malignancy [2,3]. In such cases, maintaining a high index of clinical suspicion and promptly pursuing cross-sectional imaging is critical for identifying occult extrinsic pathology and avoiding delays in diagnosis.

Establishing a definitive tissue diagnosis in mediastinal pathology remains challenging. Minimally invasive techniques such as endobronchial ultrasound-guided transbronchial needle aspiration (EBUS-TBNA) are widely utilized, but diagnostic yield may be limited by sampling error, tumor necrosis, or insufficient tissue acquisition [7]. In cases where initial biopsy results are nondiagnostic despite persistent radiographic or clinical concern, repeat tissue sampling and multidisciplinary evaluation are often necessary to establish an accurate diagnosis and guide management [5-7].

We present a case of mediastinal SCC presenting as acute dysphagia due to extrinsic esophageal compression, highlighting the diagnostic limitations of standard endoscopic evaluation and the importance of early imaging-guided and multidisciplinary diagnostic strategies.

## Case presentation

Clinical presentation 

A 69-year-old man with a history of hypertension and diabetes mellitus presented with acute onset dysphagia secondary to esophageal food impaction after ingestion of rice, beans, and meat, culminating in complete inability to tolerate oral intake or secretions and requiring emergent evaluation. Further history revealed a preceding three-to-four-month course of progressively worsening symptoms, including chronic cough, hoarseness with documented vocal cord paralysis, right upper extremity discomfort, left-sided ptosis, and an unintentional 30-40 lb weight loss, raising concern for an evolving mediastinal process prior to the acute obstructive event.

**Figure 1:**
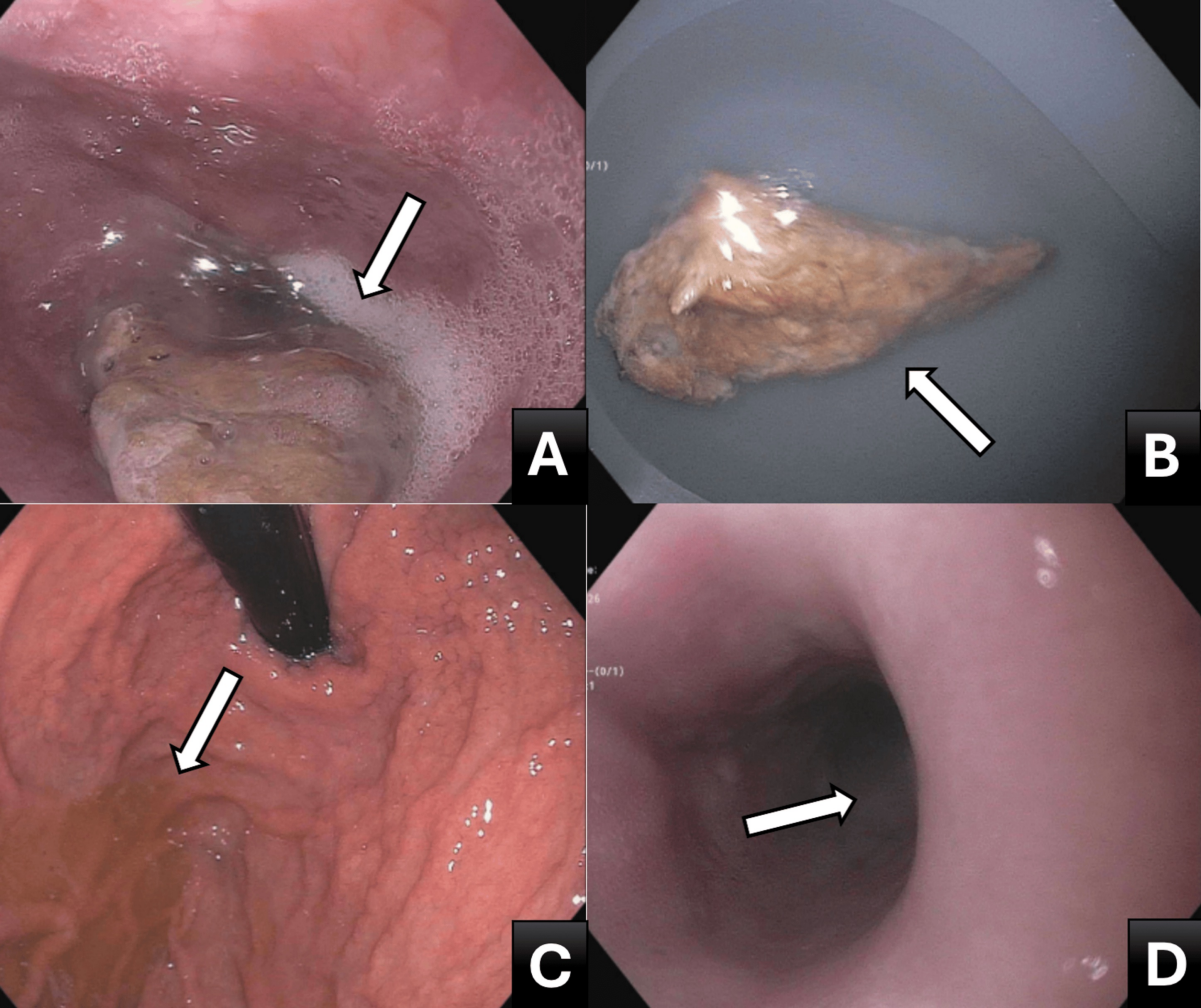
Esophagogastroduodenoscopy demonstrating food impaction and extrinsic esophageal compression without intrinsic luminal obstruction. (A) Impacted food bolus within the upper third of the esophagus. (B) Retrieved food bolus following successful endoscopic removal. (C) Gastric antrum demonstrating mildly erythematous mucosa without ulceration or mass. (D) Smooth luminal narrowing in the upper third of the esophagus, consistent with extrinsic compression in the absence of mucosal irregularity or an intraluminal lesion. These findings highlight the absence of intrinsic esophageal pathology despite clinically significant obstruction, supporting an extraluminal etiology.

Esophagogastroduodenoscopy (EGD) was performed with successful removal of the impacted food bolus from the upper third of the esophagus (Figure 1). Importantly, endoscopic evaluation did not identify any intrinsic esophageal lesion, stricture, or mucosal mass. However, significant extrinsic compression of the upper esophagus was observed approximately 24 cm from the incisors without evidence of intraluminal obstructing pathology (Figure 1), highlighting the diagnostic challenge posed by compressive mediastinal disease in the setting of a nondiagnostic initial endoscopic examination. Additional findings included mild erythematous mucosa in the gastric antrum, which was biopsied and subsequently found to be negative for malignancy. The examined duodenum was normal. Given the marked discordance between severe progressive dysphagia and the absence of intrinsic esophageal pathology, further cross-sectional imaging was pursued. Initial minimally invasive tissue sampling proved nondiagnostic, necessitating additional biopsy attempts and multidisciplinary evaluation to establish a definitive diagnosis. The patient had no known medication allergies.

Initial assessment and physical exam 

On initial assessment, the patient appeared clinically stable without evidence of acute respiratory distress or hemodynamic compromise. Despite the presence of a large mediastinal mass identified later in the diagnostic course, there were no overt physical findings suggestive of advanced mediastinal compression, including facial edema, upper extremity swelling, venous distention, or other signs concerning for superior vena cava syndrome. No palpable cervical or supraclavicular lymphadenopathy was documented. The relative absence of classic external markers of mediastinal disease contributed to the occult nature of the underlying pathology despite progressive compressive symptoms.

Laboratory evaluation demonstrated leukocytosis with neutrophil predominance and elevated inflammatory markers, suggestive of an active systemic inflammatory or malignant process. Mild normocytic anemia and hypoalbuminemia raised concern for chronic underlying disease, including malignancy and cancer-associated inflammatory burden. Collectively, the patient’s presentation demonstrated significant clinical discordance between severe progressive dysphagia and the limited external physical manifestations of mediastinal pathology.

Laboratory and diagnostic testing

Initial laboratory evaluation demonstrated leukocytosis with neutrophilic predominance, mild normocytic anemia, and intermittent thrombocytosis. Additional findings included mild hyponatremia, hypoalbuminemia, and intermittent hyperglycemia, while renal function remained preserved. Carcinoembryonic antigen (CEA) was elevated at 23.6 ng/mL, raising concern for malignancy. Infectious workup, including HIV and hepatitis C serologies, was negative. A summary of laboratory findings is presented in Table 1.

**Table 1:** Summary of Laboratory Findings at Admissions and During Hospitalization. Values at admission represent the earliest recorded laboratory measurements upon hospital presentation. Ranges during hospitalization reflect the minimum and maximum values observed throughout the inpatient course. Abbreviations: WBC = white blood cell count; CRP = C-reactive protein; CEA = carcinoembryonic antigen; PT = prothrombin time.

Laboratory Test (Unit)	Admission	Range During Hospitalization	Reference Range
WBC (×10³/µL)	11.0	9.7 – 20.1	4.0–10.0
Hemoglobin (g/dL)	10.7	10.5 – 12.0	13.5–17.5
Hematocrit (%)	32.0	31.8 – 35.7	41–53
Platelets (×10³/µL)	381	368 – 479	150–450
Neutrophils (%)	70.7	63.3 – 83.9	40–70
Absolute Neutrophils (×10³/µL)	7.8	6.2 – 16.8	1.5–7.0
Lymphocytes (%)	15.7	6.7 – 24.4	20–40
Sodium (mmol/L)	133	130 – 136	135–145
Potassium (mmol/L)	4.1	4.1 – 4.5	3.5–5.0
CO₂ (mmol/L)	22	20 – 25	22–29
Creatinine (mg/dL)	0.60	0.52 – 0.70	0.7–1.3
Glucose (mg/dL)	91	91 – 257	70–110
Albumin (g/dL)	3.3	3.3 – 3.9	3.5–5.0
CRP (mg/dL)	—	6.2	<0.5
CEA (ng/mL)	—	23.6	<3
PT (sec)	13.2	13.2 – 13.9	11–13.5

**Figure 2:**
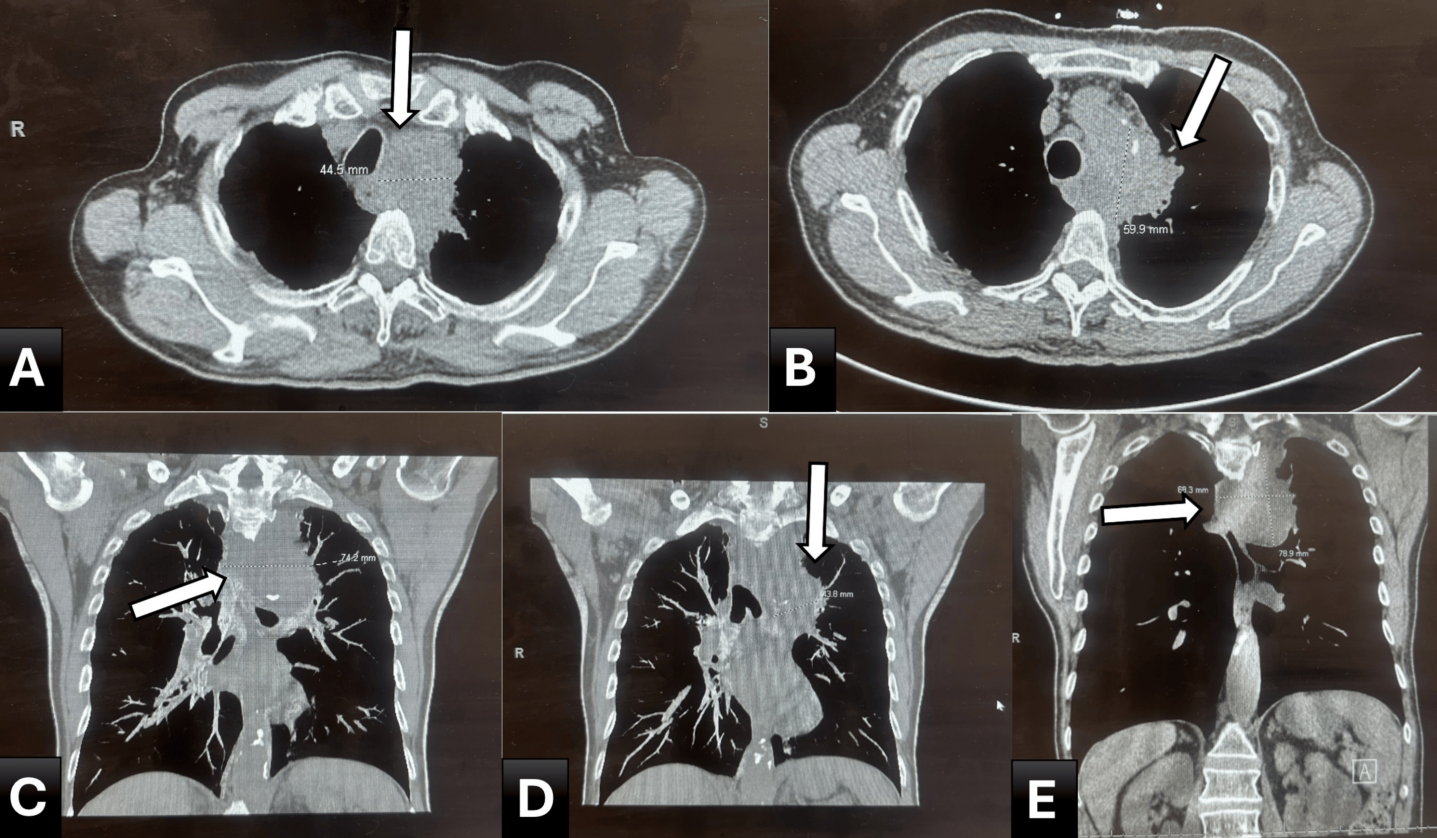
Contrast-enhanced computed tomography (CT) of the chest demonstrating a large anterior mediastinal mass (arrows). Mass visualization on axial (A, B), coronal (C, D), and sagittal (E) views. The mass measures up to approximately 7.8 cm in maximal dimension and exerts significant mass effect on adjacent mediastinal structures, including displacement and compression of the esophagus, correlating with the patient’s presenting symptom of dysphagia.

Initial imaging with chest radiograph showed chronic biapical pleural-parenchymal changes without acute abnormalities. However, contrast-enhanced computed tomography (CT) of the chest revealed a large superior mediastinal mass extending into the left lower neck and anterior mediastinum, measuring approximately 7.8 cm in maximal dimension. The mass caused significant extrinsic compression and deviation of the esophagus and demonstrated encasement of the aortic arch and its major branches (Figure 2).

CT of the abdomen and pelvis did not demonstrate definitive distant metastatic disease but revealed hepatomegaly and a benign-appearing renal cyst. CT of the brain showed no evidence of intracranial metastasis.

Given the patient’s dysphagia, EGD confirmed extrinsic compression without an intrinsic lesion. A swallow study demonstrated mechanical obstruction with esophageal deviation and focal stenosis without aspiration.

Tissue diagnosis was initially pursued through ultrasound-guided fine needle aspiration (FNA) of a right axillary lymph node, which was nondiagnostic due to insufficient cellular material. Subsequent bronchoscopy with EBUS-TBNA of the mediastinal mass and lymph node stations was also nondiagnostic, yielding predominantly blood and benign lymphoid fragments.

Given persistent high clinical suspicion, additional tissue sampling was ultimately performed, which demonstrated SCC, establishing a definitive diagnosis.

Multidisciplinary board discussion

The patient’s case was discussed in a multidisciplinary setting involving gastroenterology, pulmonology, oncology, hematology, interventional radiology, and thoracic surgery services. Initial diagnostic considerations included occult malignancy, lymphoma, metastatic disease, chronic inflammatory conditions, and infectious mediastinal pathology. The elevated CEA level raised concern for an underlying gastrointestinal or thoracic malignancy, although cross-sectional imaging favored a primary mediastinal process causing extrinsic esophageal compression.

Following nondiagnostic initial sampling with EBUS-TBNA, the multidisciplinary team debated alternative tissue acquisition strategies to obtain a definitive diagnosis. Considerations included repeat EBUS-guided sampling with larger-caliber needles, computed tomography-guided core needle biopsy, mediastinoscopy, and surgical biopsy approaches to improve tissue yield and permit more extensive histopathologic and immunohistochemical evaluation. Concern was raised that the initial nondiagnostic results may have reflected sampling error, tumor necrosis, or insufficient cellular architecture for definitive characterization.

Given the persistent high clinical suspicion for malignancy based on the patient’s progressive compressive symptoms, significant weight loss, vocal cord paralysis, and imaging findings demonstrating extensive mediastinal vascular encasement, further tissue acquisition was strongly recommended despite the initially nondiagnostic pathology. Subsequent repeat diagnostic sampling ultimately confirmed SCC, establishing the underlying etiology of the mediastinal mass and explaining the patient’s progressive dysphagia and compressive symptomatology.

Diagnosis and management 

Based on the patient’s progressive compressive symptoms, cross-sectional imaging findings, and subsequent histopathologic confirmation, the patient was diagnosed with SCC presenting as a large locally invasive mediastinal mass. Imaging demonstrated extensive tumor involvement extending from the lower neck into the superior mediastinum with circumferential encasement of major mediastinal vascular structures, including the aortic arch, carotid artery, and subclavian vessels, as well as marked compression of the proximal esophagus. The lesion was also associated with recurrent laryngeal nerve involvement manifested clinically by vocal cord paralysis and findings concerning for Horner syndrome, indicating advanced locoregional disease.

Initial management focused on stabilization and symptomatic relief, including successful endoscopic removal of the impacted esophageal food bolus via EGD. Following definitive tissue diagnosis, the case was reviewed in a multidisciplinary oncologic setting involving thoracic surgery, medical oncology, radiation oncology, pulmonology, and gastroenterology. The patient was not considered a surgical candidate because of extensive circumferential vascular encasement involving the aortic arch and supra-aortic branches, which significantly increased the risk of incomplete resection and major operative morbidity. Additionally, the degree of mediastinal invasion and proximity to critical neurovascular structures suggested unresectable locally advanced disease.

Management planning therefore shifted toward nonsurgical oncologic treatment and further staging evaluation to identify the primary source and overall extent of disease. The patient was subsequently considered for systemic therapy and radiation-based treatment approaches aimed at disease control and palliation of compressive symptoms.

Clinical outcome 

The patient experienced improvement in acute obstructive symptoms following endoscopic removal of the impacted food bolus and was able to tolerate oral intake with aspiration precautions. However, the subsequent clinical course was dominated by the diagnosis of an invasive mediastinal SCC with extensive regional vascular encasement and compressive involvement of the esophagus and adjacent mediastinal structures. Imaging demonstrated near-complete encasement of the aortic arch and involvement of multiple major mediastinal vessels, findings that precluded surgical resection.

Following tissue confirmation via EBUS-guided biopsy, the patient was discharged in stable condition with plans for outpatient multidisciplinary oncologic evaluation, including further staging and treatment planning. Definitive oncologic therapy and long-term outcomes were not available at the time of reporting.

## Discussion

Background 

Mediastinal masses were historically characterized through surgical and autopsy series, with early classification based on anatomical location and gross pathology [8]. Contemporary understanding has evolved with advances in cross-sectional imaging and molecular diagnostics, culminating in the World Health Organization (WHO) classification, which categorizes mediastinal tumors into thymic epithelial tumors, lymphomas, germ cell tumors, and mesenchymal neoplasms [8,9].

Mediastinal tumors are relatively uncommon, with reported prevalence ranging from approximately 0.7% to 1.7%, and are increasingly detected due to the widespread use of imaging modalities [10,11]. The anterior mediastinum is the most common site of primary tumors in adults, where thymic malignancies, lymphomas, and germ cell tumors predominate [9,11]. These tumors demonstrate distinct demographic patterns, with germ cell tumors more frequently affecting younger males, whereas thymic neoplasms are more commonly observed in middle-aged and older adults [9,12].

Malignant involvement of the mediastinum may also occur through metastatic disease or direct extension from adjacent thoracic structures. Among these, SCC, most commonly arising from pulmonary or upper aerodigestive tract origins, may present as a mediastinal mass through nodal conglomeration or local invasion, often manifesting with compressive symptoms rather than primary mucosal findings [9,13].

Risk factors vary by tumor subtype and include genetic syndromes such as Klinefelter syndrome in mediastinal germ cell tumors, as well as immunologic and environmental factors associated with lymphoid malignancies [8]. Mediastinal tumors exhibit a broad spectrum of biological behavior, ranging from benign cystic lesions to highly aggressive malignancies, with clinical outcomes largely determined by histologic subtype, tumor burden, and extent of local invasion [9,11].

Given their proximity to critical thoracic structures, morbidity is frequently driven by local mass effect rather than metastatic burden, necessitating a multidisciplinary, imaging-guided diagnostic approach [10,11].

Pathology/pathophysiology 

Anterior mediastinal tumors most commonly arise from thymic epithelial cells, germ cells, or lymphoid precursors; however, mediastinal involvement may also occur secondary to malignancies arising from adjacent thoracic structures [9,11]. SCC typically originates from pulmonary or upper aerodigestive tract epithelium and may involve the mediastinum through direct extension or metastatic nodal disease [9,13].

At the molecular level, SCC is characterized by alterations in tumor suppressor pathways, including TP53 mutations, and dysregulation of signaling pathways such as PI3K/AKT, promoting uncontrolled proliferation and resistance to apoptosis [13].

Tumor expansion within the confined mediastinal space results in local invasion and mass effect on adjacent structures, including the esophagus and major vascular branches [11]. In this case, extrinsic compression of the esophagus led to mechanical obstruction, manifesting as dysphagia and acute food impaction, while vascular encasement reflected locally advanced disease [9,11].

Histologically, SCC demonstrates malignant squamous differentiation with keratinization and may exhibit necrosis and fibrosis, which can limit the diagnostic yield of minimally invasive biopsy techniques [11,13]. This explains the initially nondiagnostic sampling observed in this patient.

Comparative analysis with the current literature 

*Clinical Presentation* 

Mediastinal masses are frequently asymptomatic or present with nonspecific symptoms related to compression of adjacent structures, including dyspnea, cough, chest discomfort, or dysphagia [9,11]. Dysphagia, when present, is typically progressive and associated with advanced disease or significant mass effect. In contrast, this patient presented with acute food impaction, an uncommon and potentially misleading initial manifestation that may initially suggest primary esophageal pathology rather than an underlying mediastinal process.

Additionally, while anterior mediastinal tumors encompass a broad differential diagnosis, SCC more commonly arises from pulmonary or upper aerodigestive tract origins with secondary mediastinal involvement [9,14]. The patient’s age was consistent with typical SCC demographics; however, the absence of an intrinsic esophageal lesion on endoscopy represented an atypical presentation that contributed to initial diagnostic uncertainty and delayed definitive characterization of the underlying pathology.

Diagnostic Workup

Current literature emphasizes a stepwise diagnostic approach in which cross-sectional imaging serves as the cornerstone for localization and characterization of mediastinal masses [11,14]. In this case, computed tomography appropriately identified a large invasive mediastinal mass with extensive vascular encasement and extrinsic esophageal compression, findings highly suspicious for advanced malignancy. The differential diagnosis for anterior mediastinal masses includes thymic tumors, lymphoma, germ cell tumors, and metastatic disease [9,11]. Histopathologic confirmation remains essential because imaging findings alone are often insufficient to distinguish among these entities.

This case highlights an important diagnostic limitation in current approaches to dysphagia evaluation. Initial endoscopic assessment failed to identify intrinsic esophageal pathology despite severe obstructive symptoms, creating a potential diagnostic blind spot that could have delayed recognition of the mediastinal process. Furthermore, although minimally invasive techniques such as EBUS-TBNA are widely utilized for tissue acquisition, diagnostic yield may be limited by tumor necrosis, fibrosis, or sampling error [9,15]. In this patient, multiple appropriately targeted biopsy attempts remained nondiagnostic despite extensive radiographic evidence of invasive disease.

The prognostic implications of this delay are clinically significant in the setting of locally advanced SCC. Prolonged diagnostic uncertainty may permit continued tumor progression, worsening vascular encasement, increasing mediastinal invasion, and further compromise of surgical candidacy before initiation of definitive oncologic therapy. This case therefore emphasizes that nondiagnostic biopsy should not be interpreted as reassuring when imaging and clinical findings remain highly suspicious for malignancy. Early consideration of alternative tissue acquisition strategies, including repeat sampling, core needle biopsy, mediastinoscopy, or surgical biopsy, may be necessary to avoid delays in diagnosis and treatment initiation.

*Management* 

Management of mediastinal malignancies is highly dependent on histologic subtype, staging, and resectability [9,13]. For SCC involving the mediastinum, treatment typically consists of systemic therapy, radiation therapy, or multimodal oncologic management, particularly in patients with locally advanced unresectable disease.

In this case, management appropriately prioritized acute symptom relief through endoscopic removal of the impacted food bolus, followed by urgent imaging and attempts at tissue diagnosis. However, unlike more straightforward presentations in which diagnosis is rapidly established, definitive oncologic management was delayed by repeated nondiagnostic sampling despite persistent radiographic concern for aggressive malignancy. Imaging demonstrated near-complete encasement of the aortic arch and involvement of major mediastinal vessels, findings that significantly limited surgical options and suggested advanced locoregional invasion at the time of diagnosis.

This case underscores how delays in tissue confirmation may directly influence treatment timelines and potentially alter prognosis in invasive mediastinal SCC. Persistent multidisciplinary reassessment and early escalation to alternative biopsy strategies may therefore be critical when initial minimally invasive approaches fail to establish a diagnosis.

*Clinical Outcome* 

Prognosis in mediastinal SCC varies according to primary origin, stage, extent of local invasion, and response to therapy, with locally advanced disease generally associated with poorer outcomes [13,16]. Although the patient experienced immediate symptomatic improvement following endoscopic intervention, the broader clinical course was ultimately defined by the presence of a large invasive mediastinal malignancy with extensive vascular encasement [13,16].

At the time of reporting, long-term oncologic outcomes were not yet available. However, the degree of mediastinal invasion and delayed definitive diagnosis raised concern for aggressive locoregional disease progression prior to initiation of oncologic therapy. This further emphasizes the prognostic importance of timely tissue diagnosis in patients with highly suspicious mediastinal masses.

Literature Summary

Existing literature generally describes a relatively linear diagnostic pathway in mediastinal masses, where imaging is followed by successful tissue diagnosis and prompt initiation of therapy [9,11]. This case deviates from that paradigm by illustrating a clinically significant scenario of persistent diagnostic uncertainty despite appropriate imaging and repeated biopsy attempts.

Although limitations of minimally invasive sampling techniques are recognized, their potential impact on prognosis and treatment delay is often underemphasized in clinical practice [9,15,16]. In this patient, nondiagnostic biopsy was not incidental but central to the clinical course, contributing to delayed definitive diagnosis in the setting of a locally advanced invasive malignancy. The case therefore reinforces that inconclusive pathology should not diminish clinical suspicion when radiographic and symptomatic findings strongly suggest aggressive disease.

Furthermore, presentation as acute food impaction highlights an uncommon manifestation of mediastinal malignancy and underscores the importance of considering extrinsic mediastinal compression when endoscopic findings are incongruent with the severity of dysphagia symptoms.

What we learned from this case 

This case challenges the traditional, lumen-centered approach to dysphagia by demonstrating that clinically significant obstruction may arise entirely from extraluminal disease, even in the absence of intrinsic esophageal pathology. It highlights a critical diagnostic blind spot: when endoscopic findings are negative or discordant with symptom severity, the underlying pathology may reside outside the field of direct visualization.

A key insight is the concept of diagnostic asymmetry between modalities. Endoscopy provides high-resolution evaluation of the mucosal surface but is inherently limited in assessing external compression. In contrast, cross-sectional imaging not only detects extraluminal pathology but also characterizes its biological behavior through features such as vascular encasement and tissue invasion. This case reinforces that dysphagia should be approached as an anatomical problem rather than a purely luminal one, particularly when initial evaluation is inconclusive.

Perhaps most importantly, this case reframes nondiagnostic biopsy as a clinically meaningful finding rather than a procedural failure. In aggressive malignancies such as SCC, tumor heterogeneity, necrosis, and architectural disruption may preferentially yield nonviable or nonrepresentative tissue. Thus, repeated nondiagnostic sampling should heighten, rather than diminish, suspicion for malignancy when supported by imaging and clinical context.

This case also introduces the principle of anatomy-driven biopsy strategy, where sampling targets should prioritize the dominant disease burden rather than the most accessible site. Early escalation to alternative modalities, including core biopsy or surgical approaches, may be necessary to obtain diagnostic tissue and avoid delays in care.

Finally, this case emphasizes that morbidity in mediastinal malignancies is often driven not by metastatic spread, but by local biomechanical effects, including compression of the esophagus and great vessels. Recognizing this mechanism expands the clinical understanding of how malignancy can present and underscores the importance of integrating structural, radiologic, and pathologic data in complex diagnostic scenarios.

## Conclusions

This case underscores that dysphagia should not be reflexively attributed to intrinsic esophageal pathology, particularly when endoscopic findings are incongruent with clinical severity. A normal or near-normal luminal evaluation does not exclude clinically significant disease, and reliance on endoscopy alone may delay diagnosis when the underlying process is extraluminal. In this patient, cross-sectional imaging was pivotal in identifying a large invasive mediastinal mass, ultimately diagnosed as SCC, responsible for esophageal compression and symptomatology. Accordingly, early cross-sectional imaging should be strongly considered in patients presenting with acute or progressive dysphagia when initial endoscopic evaluation fails to identify a clear intraluminal cause, particularly in the presence of systemic symptoms, weight loss, or compressive features suggestive of mediastinal pathology.

Beyond the diagnostic sequence, this case highlights a broader clinical principle: diagnostic discordance between modalities should prompt escalation rather than reassurance. Repeatedly nondiagnostic biopsy results, when interpreted in isolation, may obscure the presence of aggressive malignancy. However, when integrated with imaging and clinical context, they may instead reflect tumor heterogeneity, necrosis, or sampling limitations and should prompt consideration of alternative tissue acquisition strategies.

Ultimately, this case emphasizes that accurate diagnosis in complex presentations requires an anatomy-driven, multimodal approach with careful alignment between clinical findings, imaging, and pathology. Recognizing extraluminal causes of dysphagia and reframing nondiagnostic biopsy as a meaningful clinical signal rather than a reassuring finding may help prevent avoidable diagnostic delays and improve management of patients with invasive mediastinal malignancies such as SCC.
